# Analysis of Selected Spark Plasma Sintering Parameters on the Mechanical Properties of Sintered X30Cr13 Steel

**DOI:** 10.3390/ma18133084

**Published:** 2025-06-29

**Authors:** Anna Kulakowska, Teresa Bajor, Anna Kawalek

**Affiliations:** 1 Faculty of Science and Technology, Jan Dlugosz University in Czestochowa, 42-201 Czestochowa, Poland; a.kulakowska@ujd.edu.pl; 2 Faculty of Production Engineering and Materials Technology, Czestochowa University of Technology, 42-201 Czestochowa, Poland; teresa.bajor@pcz.pl

**Keywords:** spark plasma sintering (SPS), steel powder, sintering powder, holder screws

## Abstract

This paper presents the possibilities of using the reaction sintering method for the production of tool steel used in medicine. The applied method enables the sintering of powders in one technological process. The SPS (spark plasma sintering) process is a technology in which electric pulses generate heat and pressure, which allows for the quick and effective connection of powder particles into a homogeneous material with high density and good mechanical properties. As a result, a product of small dimensions and a precisely defined chemical composition, established at the stage of preparing the powder mixture, is obtained. The advantages of the applied production process are the sintering time and small amounts of post-production waste compared to conventional methods of producing a finished product from steel. The method of producing a semi-finished product is particularly useful in the case of small-scale and small-sized production. The subject of the research was the analysis of the conditions for obtaining X30Cr13 martensitic steel used for the production of surgical instruments. This paper analyzes the effect of sintering temperature and time on sinterability and on selected physical and mechanical properties of the obtained materials. The sintering parameters of the starting mixture have been optimized to obtain the highest possible sinter properties, such as density and hardness. Based on the analysis of the results, it was found that the powder preparation method for the SPS process and the grain size significantly affect the microstructure and mechanical properties of the final product. The optimal sintering parameters for X30Cr13 steel are a temperature of 950 °C and a sintering time of 12 min. Furthermore, the use of the SPS method allows for a reduction in the manufacturing costs of martensitic steel semi-finished products.

## 1. Introduction

Iron-based alloys have been of great interest to many research centers for many years due to their very good mechanical properties and high corrosion resistance in various environments. These properties are essential when manufacturing products that are used in many industries. One area of application for corrosion-resistant steels in moderately aggressive environments is the manufacture of surgical products/tools [1,2,3,4,5].

Obtaining these materials using conventional methods is not a problem for many entrepreneurs, but an innovative solution may be to obtain materials with the same chemical composition but significantly higher strength properties using the Spark Plasma Sintering (SPS) method.

The process of consolidating powder materials through the use of spark plasma sintering (SPS) makes it possible to achieve success in this area. The spark plasma sintering (SPS) method has been successfully used in the manufacture of metals and their alloys that are difficult to achieve with conventional sintering methods. The SPS method uses pulses of direct current to induce an immediate local rise in temperature in micro-areas, and then the sintered material particles are consolidated. Thermal energy is released simultaneously throughout the volume of the sintered material, indicating the energy efficiency of this method due to the low loss of thermal energy to the environment [6]. The solution used raises a lot of controversy among researchers, especially when analyzing the change occurring inside sintered powder materials. In the literature, one can find different opinions regarding the presence of plasma in the conducted sintering process [7,8,9,10,11,12,13]. Nevertheless, the spark plasma sintering method has found a lot of application for sintering a wide range of materials [14,15].

The use of the SPS method makes it possible to obtain a material with very good properties that can be used for small batch production of connecting elements in surgical instruments [16]. Among others, materials with high mechanical properties based on Fe alloys are obtained using the indicated method. In addition, the use of heat treatment in the form of quenching and tempering allows the material to have the hardness of 50HRC.

An additional advantage of using the SPS method is the possibility of sintering powders obtained from technological waste (blanks, moldings, stampings) of specific steel grades.

Examples include the results of sintering high-speed steels (HSS) presented in this paper [17,18,19,20]. The obtained sinters with a powder granulation of <45 μm have a density close to theoretical density (99.5%). The best effect was achieved with the following parameters: T = 1100 °C, t = 5 min and the pressure of 60 MPa.

With its advantages, the SPS method can be used to obtain semi-finished products from metal powders with high melting points, such as Nb, as well as the ones whose traditional manufacturing technology is characterized by long production cycles and significant losses of material during their surface machining, such as Nb and Zr [21,22,23].

The paper [24] presents results of the research on the process of sintering a FeMo alloy. It has been shown that it is possible to achieve 100% theoretical density after sintering at temperatures above 835 °C and powder granulation above 490 nm. Articles [25,26,27] present the results of research on obtaining components from highly dispersed ODS Fe-14%Cr steel used in nuclear reactors, which are characterized by a large amount of chromium in their chemical composition. Due to its chemical composition, such a material is sintered at temperatures from 900 °C to 1050–1150 °C in 2 to 10 min at a pressure of 60–70 MPa. The indicated parameters allow one to obtain a material with the minimum density of 98% [28,29,30,31]. In the case of steel 316 L, the sintering process is carried out at temperatures above 1000 °C, and the achieved sinter density is 98–99% of the theoretical density [32,33].

The use of the SPS method allows for freedom of configuration of the feedstock material. An example is the Y_2_O_3_ oxide, which is often added to steel to strengthen it. This allows for the use of these steels in very harsh working conditions where conventional structural materials cannot be used. It is also possible to use the discussed method to produce sinters by adding nanotubes to steel [34,35,36] or combination, for example, with 2205 duplex stainless steel [37]. It offers the possibility of reducing costs of materials by preparing a small amount of batch material, which is very advantageous in the manufacture of parts for the production of materials with special parameters for special applications, for example in the production of connecting elements for surgical instruments [16].

X30Cr13 steel is often used for screws for surgical needle holders. Although these instruments are not exposed to a highly corrosive environment for long periods of time, regular sterilization cycles at high temperatures accelerate the wear and tear of these components. In addition, the screws in the needle holders must be of high hardness since they carry relatively high loads in relation to their small dimensions.

## 2. Purpose of Work

The purpose of this paper was to determine the parameters of the process of SPS sintering of the X30Cr13 martensitic steel powder. An analysis was carried out to assess the effects of temperature and sintering time on sinterability and on selected physical and mechanical properties of the obtained semi-finished products.

Due to the miniature size of these fasteners, the production of needle holder screws requires surprisingly little material—even in high-volume production, steel consumption does not exceed a few kilograms per year. This specificity makes the SPS (spark plasma sintering) method likely to be the optimal technological choice. This method enables rapid consolidation of metallic powders using pulses of electric current and pressure, which translates into short production times, its economic viability in small batches, and the ability to precisely control the chemical composition of the material. This makes it possible to obtain a steel with properties ideally suited to the requirements for surgical needle holder components.

## 3. Research Material and Methods

A commercial powder with a granularity of less than 10 μm obtained from steel wire with a suitable chemical composition and a blend prepared in-house with a granularity of less than 45 μm were used to produce the semi-finished X30Cr13 martensitic steel. The chemical composition of the tested samples was in accordance with EN 10088-1:2014 [38] for X30Cr13 steel (Table 1).

The powder blends were prepared in a Turbula T2F-type mixer (Tomasova Lea S.R.O., Pardubice, Czech Republic). The mixing time of the commercial blend was 30 min, and the mixing time of the blend prepared in-house was about 2 h.

The sintering process was carried out using the SPS 10-4 (Thermal Technology LLC, Minden, NV, USA) device, owned by the Department of Production Engineering and Materials Technology at the Czestochowa University of Technology.

For sintering, tools made of graphite of grade 2333 (Mersen, Paris, France) were used. The feed chamber in the graphite tool assembly was filled with a powder blend. The graphite film separating the powder from the tools was used for technological reasons. Its application facilitates the removal of the sample from the die after the sintering process. Papyex N998 graphite film (Mersen, Paris, France) was placed between the powder blend and the die and stamps for technological reasons. Its application facilitates the removal of the sample from the die after the sintering process. The tools prepared this way were placed in a sintering chamber to conduct sintering, under vacuum conditions.

Prior to sintering, the prepared powder blends were examined using a URD-6 (U.R.D., Tokyo, Japan) polycrystalline materials X-ray diffractometer and a Rigaku Mini Flex D-600 (Rigaku, Tokyo, Japan). X-ray diffraction (XRD) measurements were performed employing Cu Kα radiation (λ ≈ 1.54 Å) in continuous 2θ/θ scanning mode over the 10–90° 2θ range, with a K_β_ filter and standard slits (IHS, DS, SS, RS), and detection was carried out using a D/teX Ultra2 detector (Rigaku, Tokyo, Japan).

The density of the sinters was measured using the Archimedes’ immersion method in accordance with the PN-EN ISO 2738:2001 standard according to dependency, as described in Equation (1) [39]. The sample was first weighed in air. Then, fully saturated pores were filled with demineralized water, and the sample was weighed again while immersed in the same liquid. The difference in mass was used to calculate the sample volume and density. Measurements were performed using a Ohaus analytical balance (Ohaus, Axis, Mettler Toledo) equipped with a dedicated density determination kit for solids, liquids, and gases.

(1) $$g=\frac{m_{1\;}\times g_{w}}{m_{3}-m_{4}}$$

where

*m*_1_—dry sample mass,
*m*_3_—mass of sample weighed above water after soaking,
*m*_4_—mass of sample weighed in water after soaking,
*g_w_*—water density, dependent on ambient temperature.

Microscopic observations of the structures after the sintering process were carried out using a Nikon MA 200 optical microscope (Nikon Corporation, Tokyo, Japan). The samples were prepared by grinding, polishing, and chemical etching to reveal the microstructure. Observations were performed under bright-field illumination at various magnifications.

The microstructure of the powders and sintered samples after normalization were examined using a scanning electron microscope (SEM) with an EDS analyzer (Tescan, Brno, Czech Republic). Microscopic observations were performed using a Tescan Vega 3 SBU scanning electron microscope (Tescan, Brno, Czech Republic) equipped with an Oxford Instruments X-act EDS system (Tescan, Brno, Czech Republic). SEM images were acquired at an accelerating voltage of 5 kV and 1000× magnification and using a secondary electron (SE) detector. Chemical composition analyses using EDS were conducted at 20 kV, using a backscattered electron (BSE) detector, with a working distance (WD) below 15 mm and a beam intensity (BI) of 17. Aztec 2.1 software was used for data acquisition and analysis.

The samples examined were standard metallic iron-based alloys, which allowed for SEM measurements without the need for a conductive coating and enabled the use of typical measurement conditions.

The hardness of the resulting sinter was measured using the Vickers method with a FutureTech FM-700 hardness tester (load: 4.9 N, dwell time: 10 s), produced by Future Tech Corporation, Kawasaki-City, Japan. Ten measurements were taken for each sample.

## 4. Analysis of Test Results

**Figure 1:**
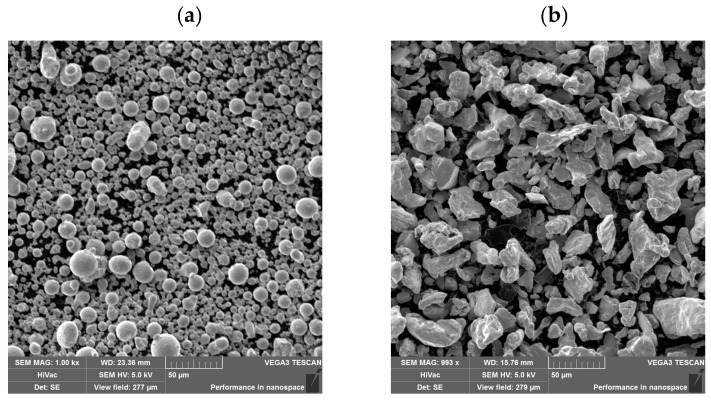
Microstructure of powders prepared for sintering: (**a**) commercial blend; (**b**) proprietary blend.

Before sintering, the powders were examined using a scanning electron microscope (SEM) equipped with an EDS analyzer. Results of tests of the commercial blend and the proprietary blend are shown in Figure 1.

**Figure 2:**
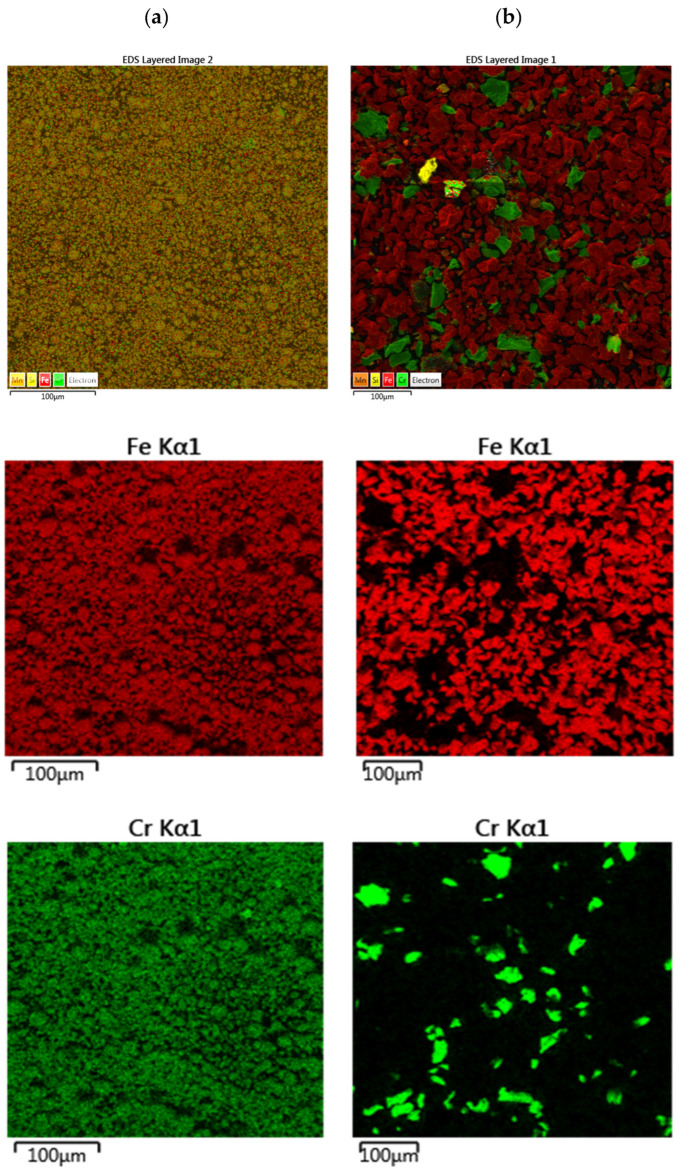
Distribution of elements in (**a**) commercial and (**b**) proprietary blends.

The powder grains of the commercial blend (Figure 1a) are characterized by a regular spherical shape of varying sizes, indicating that the powder was prepared using pulverization. In turn, the shape of the proprietary blend powder (Figure 1b) shows that the powder was prepared by grinding, and its components are significantly larger compared to the commercial blend powder. The distribution of elements in the studied powders is shown in Figure 2.

The results of the EDS analysis (Figure 2) showed an even distribution of elements throughout the tested area of the commercial blend, which is also confirmed by the Fe and Cr distribution maps. This distribution suggests that the iron grains in this blend are already saturated with the other components, such as chromium, manganese, and silicon. The distribution of elements in the proprietary blend shows clearly distinct grains of the respective elements and components of the blend. Separate grains of iron, chromium, and other components can be seen, which is natural, since no processes were carried out after the blend was prepared that could lead to the formation of any intermetallic compounds.

**Figure 3:**
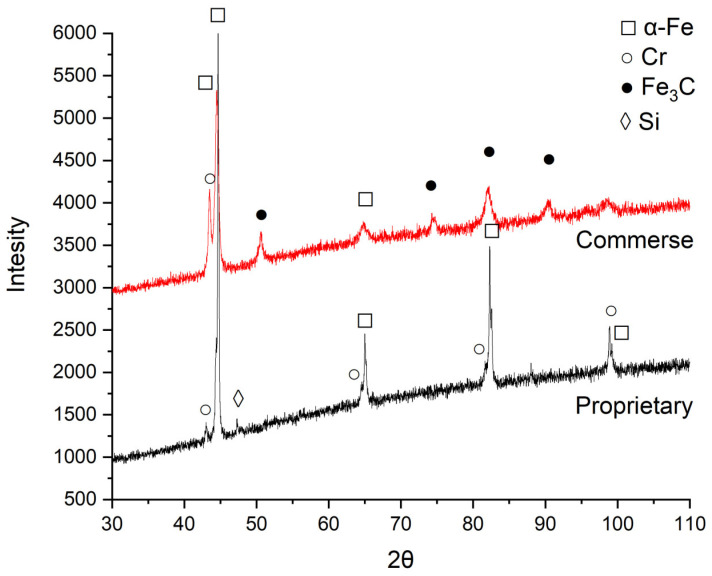
X-ray diffractions for commercial and proprietary blends prepared for sintering.

An X-ray analysis, shown in Figure 3, was performed to identify the phases in the composition of the powders.

X-ray diffractions for the proprietary blend showed intense distinct Cr and α-Fe peaks, indicating that there are powders of pure elements in the composition. The 2θ angles for these elements are very close, as can also be seen in Figure 3. Although the Si content is very low, a peak was also observed for this element.

Compared to the X-ray spectrum of the proprietary blend, the presence of Fe_3_C peaks, probably enriched with Cr, was observed for the commercial blend, which can be inferred from their shape. A peak characteristic of Fe with chromium was also observed, the shape of which suggests a high chromium content in α-Fe. Based on the results, it can be concluded that the structure of commercial powder contains ferrite and iron carbide with a high chromium content.

In order to determine the sintering temperature, preliminary tests were carried out for a blend of powders prepared in-house in the sintering temperature range of 900–1000 °C. A constant heating rate of 100 °C/min and a constant pressing pressure of 50 MPa were used. The pulse duration was 15 ms, and the duration of the interval between pulses was 20% of the pulse duration.

**Figure 4:**
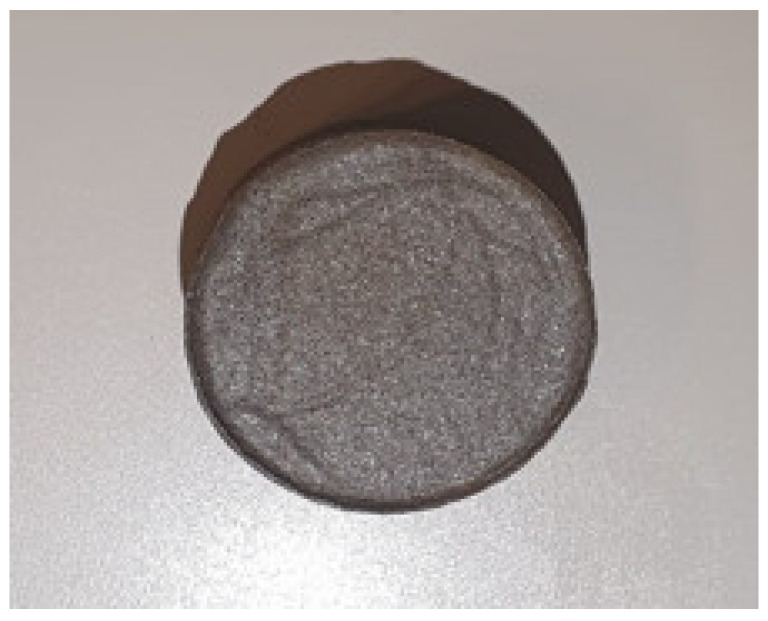
View of the sample obtained after the SPS sintering process.

As a result of the sintering process, Ø20 × 10 mm samples were obtained (Figure 4). The results of density measurements of the samples after the sintering process are shown in Table 2.

The data presented in Table 2 show that increasing the sintering temperature above 950 °C does not increase the density of the sample. For economic reasons, the optimal sintering temperature was found to be 950 °C, and this temperature was used for all the samples.

The process sintering both blends was carried out at 950 °C and at a pressure of 50 MPa. The variable parameter was sintering time, which changed in the range of 10–14 min. The sintering parameters for both powder blends are shown in Table 3.

**Figure 5:**
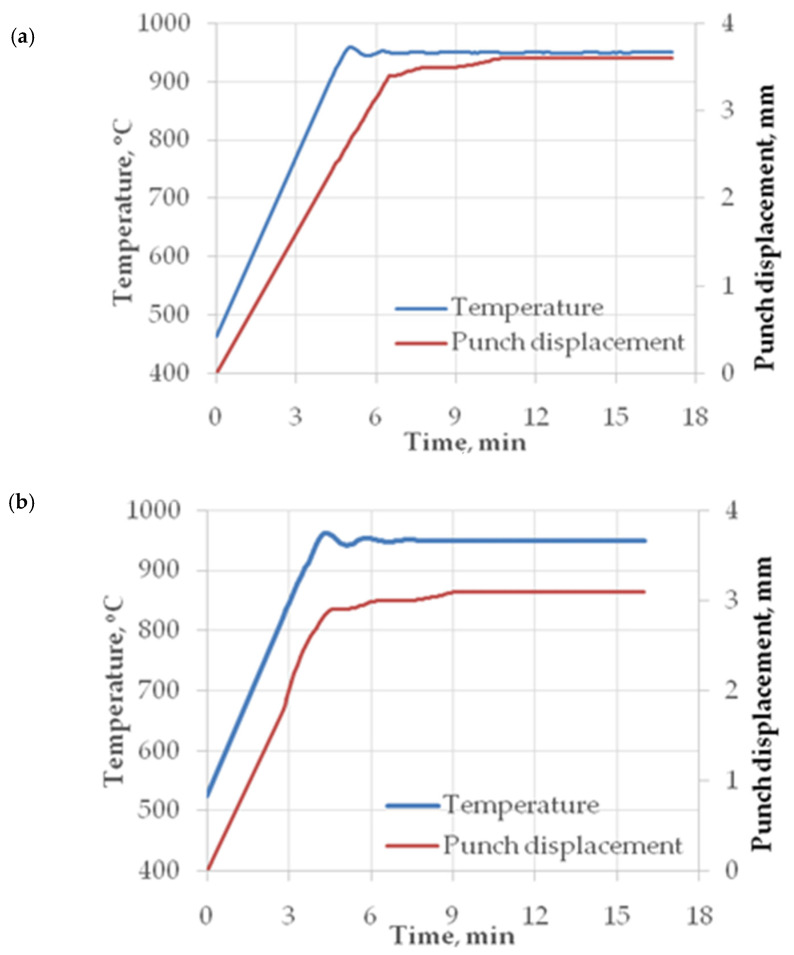
Relationships of temperature change and punch displacement recorded while sintering the samples using SPS: (**a**) proprietary blend, (**b**) commercial blend.

Examples of the temperature change and punch displacement relationships recorded during the sintering of powder blends using the SPS method are shown in Figure 5.

From the data shown in Figure 5, it can be seen that when the sintering temperature reaches 950 °C, a plateau effect can be observed on the curves showing the densification process. This indicates that the sintered powder is fully densified. The time to achieve this effect depends on the size of the powder particles. The smaller the powder particles are, the faster the consolidation.

**Figure 6:**
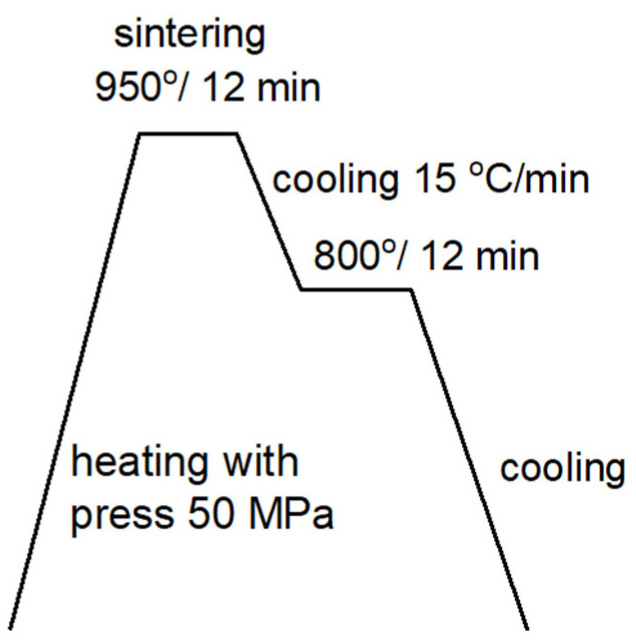
Diagram of two-stage sintering.

In order to increase the density of the sample made from the proprietary blend, a sintering process was carried out according to the diagram shown in Figure 6.

The sintering process shown in Figure 6 resulted in a sample density of 7.358 g/cm^3^, which is 93% of the theoretical density for this steel.

After the sintering process, density measurements were carried out, the results of which are shown in Table 4.

From the data presented in Table 4, it can be seen that increasing the sintering time had very little effect on increasing the density of the samples obtained. The sinters produced at 950 °C from the commercial blend have a density of 7.7 g/cm^3^, which is 97.6 percent of the theoretical density determined from the chemical composition of the powder, which is 7.884 g/cm^3^.

Sinters were made at 950 °C from a proprietary blend. The difference in the density value of sinters made from a commercial blend and a proprietary blend is due to the differences that exist in the chemical composition of each blend and the powder granulation.

**Figure 7:**
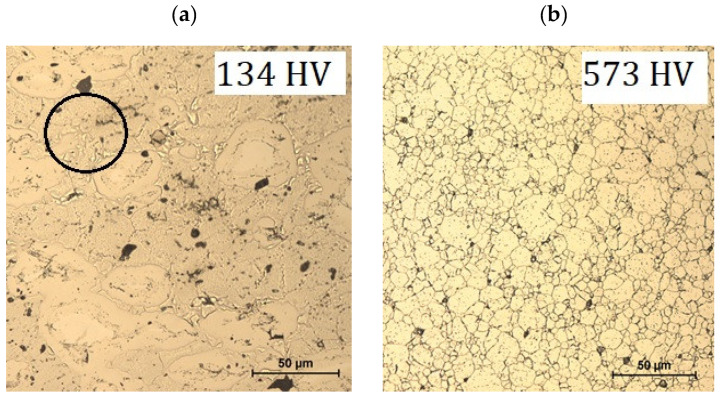
Microstructure of the samples sintered at 950 °C for 12 min from a proprietary (**a**) and commercial blend (**b**).

Figure 7 shows optical microscope images and hardness values of X30Cr13 steel samples obtained using spark plasma sintering with a proprietary blend (Figure 7a) and a commercial blend produced by grinding a wire of the said alloy prepared earlier using the traditional method (Figure 7b).

The microstructures of the samples after the SPS process using proprietary and commercial blends show certain differences. Figure 7a shows the heterogeneous structure of the metal with distinct areas of more-or-less revealed structural components. Areas of granular structures are also observed (marked in the figure). Figure 7b shows a homogeneous granular structure of the metal with the presence of precipitates evenly distributed in the solid solution. Hardness is at the reference value for this grade of steel.

The significant differences in the structure and properties of the samples shown in Figure 7a,b can be explained by the way the powder was prepared for sintering using the SPS method.

The X30Cr13 steel grade has the following composition: C, 0.26–0.35%; Cr, 12–14%; Mn, up to 1.5%; Si, up to 1%; P, up to 0.04%; and S, up to 0.015%. This grade of steel is produced using traditional metallurgical processes when chemical elements form interstitial and substitutional solid solutions. Subsequent formation and heat treatment processes ensure the uniformity of the structure and properties of the finished product. The resulting sintering powder and each of its fractions from a specific grade of steel has a formed metal crystal structure with interstitial and substitutional atoms of specific chemical elements. Therefore, the consolidation of such a powder using the SPS method is characterized by a homogeneous structure and properties at the standard level for this alloy.

**Figure 8:**
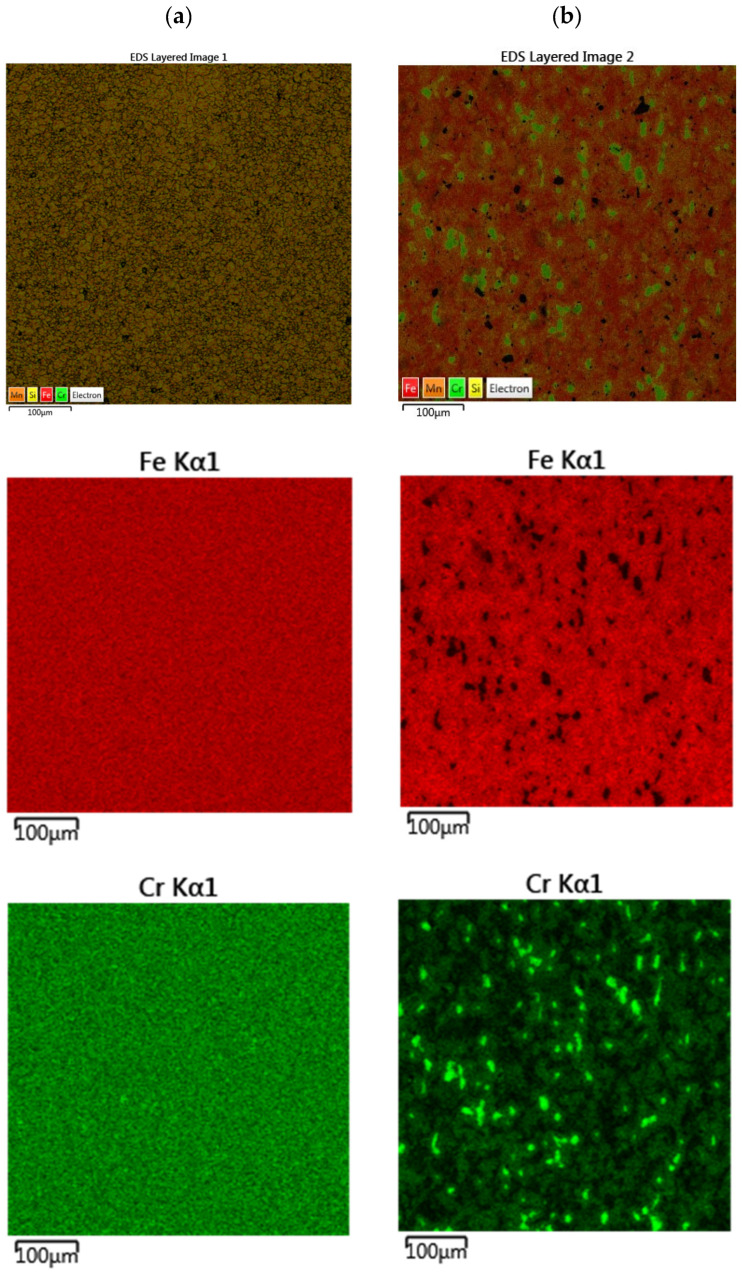
Elemental distribution in a sintered (**a**) commercial blend and (**b**) proprietary blend.

The method of preparing a proprietary blend of powders from powders of the respective chemical elements is apparently not optimal for consolidation using the SPS method. This is due to the absence of a liquid phase throughout the sintered sample, the inhomogeneous temperature field, and the lack of mixing in the SPS sintering process. Sintering results in a metal structure with heterogeneous chemical composition in local areas, which leads to a reduction in mechanical properties, including in hardness. Also visible are pores and inclusions, which are Si and Mn particles. This is confirmed by the EDS analysis as shown in Figure 8.

The microstructure observations made confirm the results of powder tests conducted before the sintering process.

**Figure 9:**
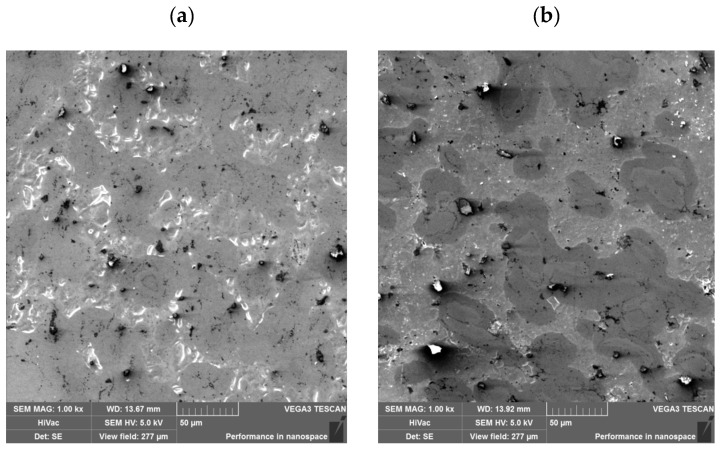
Microstructure of the samples after normalizing **(a)** sample from a proprietary blend sintered at 1000 °C for 12 min and (**b**) two-stage sintering.

**Figure 10:**
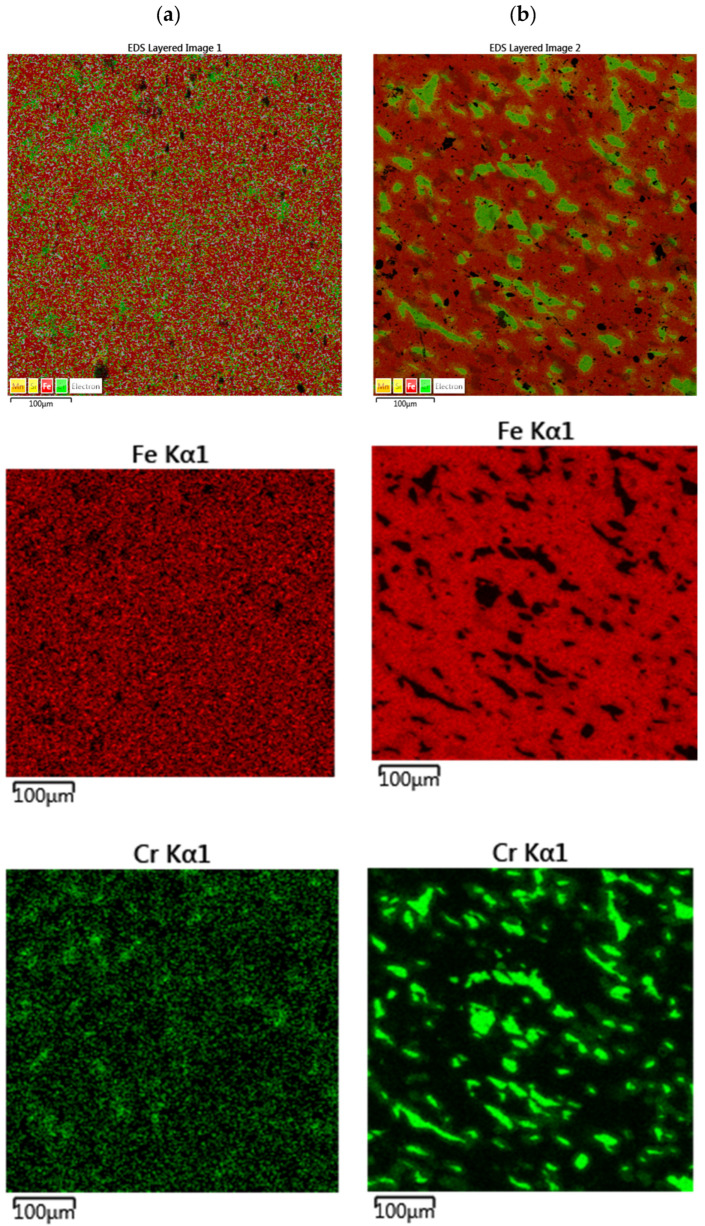
Elemental distribution of self-mixed sinters after (**a**) normalization and (**b**) two-stage sintering.

In order to standardize the microstructure of the proprietary blend sinter, a normalization process was carried out at 1000 °C for 30 min and with subsequent cooling in air. The microstructure of the samples after normalization and after two-stage sintering is shown in Figure 9. In turn, the EDS analysis is shown in Figure 10.

Based on the microstructure analysis (Figure 9a), it can be concluded that the normalization process carried out after sintering did not have a significant effect on the microstructural composition, while the EDS analysis (Figure 10a) showed a certain degree of uniformity in the distribution of Cr in Fe, as indicated by the distribution of Cr in the study area compared to the sample not subjected to normalization (Figure 9b). In turn, two-stage sintering did not result in a more uniform structure or a more uniform distribution of elements in it, compared to the proprietary blend sample sintered for 12 min (Figure 9b and Figure 10b).

The hardness of the resulting sinters was then measured using the Vickers method at a load of 4.9 N. The results of hardness measurements and hardness of the X30Cr13 steel according to EN 10088-2:20014 [40] are shown in Table 5.

As can be seen from the data presented in Table 5, the hardness of the samples made from the commercial blend averaged 556 HV. Such hardness corresponds to the hardness of this steel after the hardening process according to the relevant standard, although no martensite was observed in the microstructure. The high level of hardness of the sample may be due to the presence of Cr-enriched carbide Fe_3_C, as shown by X-ray studies of the commercial blend powder and microstructure studies of the finished samples. Samples made from the proprietary blend demonstrate a hardness of 141.33 HV. This is a much lower hardness compared to the hardness of the X30Cr13 steel of 235 HV according to the standard. The reason for this is the lack of structural transformation related to the way the proprietary blend is produced. Performing the process of normalization on the proprietary blend sample increased the hardness by 30 HV to 174 HV. In contrast, additional annealing during two-stage sintering did not increase the microhardness of the sample.

## 5. Conclusions

Based on the research, the following final conclusions were drawn:

1. The way the powder is prepared for the SPS sintering process as well as the grain size have a significant impact on the structure and mechanical properties of the final product.
2. The commercial blend, derived from steel produced using conventional metallurgical processes, exhibited a well-formed crystalline structure. The proprietary blend showed a non-uniform microstructure and local chemical inhomogeneities, which negatively affected the mechanical properties of the sintered samples.
3. The sintering temperature was selected based on density measurements of the obtained samples. The sintering time was the variable parameter, ranging from 10 to 14 min.
4. The optimal sintering process parameters for the X30Cr13 steel are a sintering temperature of 950 °C and sintering time of 12 min.
5. Additional heat treatment of the proprietary blend sample in the form of normalization at 1000 °C for 30 min resulted in a more uniform distribution of Cr in Fe and an increase in hardness by 30 HV.

The new SPS sintering technology for the manufacture of semi-finished X30Cr13 steel features a much shorter production cycle compared to the traditional method. In addition, the use of the SPS method makes it possible to reduce the cost of semi-manufacturing due to the following:

- The sintering powder can be sourced from waste material generated after processes of manufacturing finished products from the X30Cr13 steel.
- The process time is reduced.
- The waste associated with mechanical processing is eliminated.

## Figures and Tables

**Table 1:** Chemical composition of powder blends of X30Cr13 steel, %.

Blend	C	Cr	Mn	Si	Fe
Proprietary	0.31	14	0.6	0.5	84.59
Commercial	0.35	14	1.0	1.0	83.65
X30Cr13	0.26–0.35	12–14	<1.5	<1	rest

**Table 2:** Results of density measurements of samples after sintering at different temperatures.

Sintering Time, min	Sintering Temperature, °C	Density g/cm^3^
10	900	7.34
10	950	7.36
10	1000	7.36

**Table 3:** Applied sintering parameters for the examined powders.

Sintering Temperature, °C	Sintering Time, min	Pressure of Sintering/ Pressing, MPa	Atmosphere
950	10	50	Vacuum
950	12	50	Vacuum
950	14	50	Vacuum

**Table 4:** Results of density measurements of the finished samples.

Sintering Time, min	Commercial Blend	Proprietary Blend
	Density, g/cm^3^ (Relative)	
10	7.647 (0.97)	7.366 (0.93)
12	7.659 (0.97)	7.386 (0.93)
14	7.674 (0.97)	7.448 (0.94)

**Table 5:** Hardness of the tested samples.

	Sample	HV Hardness
1.	From a commercial blend	556 ± 20.9
2.	From a proprietary blend	141.33 ± 5.9
3.	From a proprietary blend after normalization	174.1 ± 4.5
4.	After sintering with additional annealing	133.8 ± 2.3
5.	X30Cr13 according to standard [40]	235 (annealed)
		450–550 (quenched and tempered)

## Data Availability

The original contributions presented in this study are included in the article. Further inquiries can be directed to the corresponding author.
